# The Effects of Objective Push-Type Sleep Feedback on Habitual Sleep Behavior and Momentary Symptoms in Daily Life: mHealth Intervention Trial Using a Health Care Internet of Things System

**DOI:** 10.2196/39150

**Published:** 2022-10-06

**Authors:** Hiroki Takeuchi, Kaori Suwa, Akifumi Kishi, Toru Nakamura, Kazuhiro Yoshiuchi, Yoshiharu Yamamoto

**Affiliations:** 1 Graduate School of Education The University of Tokyo Tokyo Japan; 2 Graduate School of Medicine The University of Tokyo Tokyo Japan; 3 Precursory Research for Embryonic Science and Technology Japan Science and Technology Agency Saitama Japan; 4 Graduate School of Engineering Science Osaka University Osaka Japan

**Keywords:** wearable activity monitor, smartphone app, sleep feedback, ecological momentary assessment, stabilized sleep timing, mood and physical symptoms

## Abstract

**Background:**

Sleep is beneficial for physical and mental health. Several mobile and wearable sleep-tracking devices have been developed, and personalized sleep feedback is the most common functionality among these devices. To date, no study has implemented an objective push-type feedback message and investigated the characteristics of habitual sleep behavior and diurnal symptoms when receiving sleep feedback.

**Objective:**

We conducted a mobile health intervention trial to examine whether sending objective push-type sleep feedback changes the self-reported mood, physical symptoms, and sleep behavior of Japanese office workers.

**Methods:**

In total, 31 office workers (mean age 42.3, SD 7.9 years; male-to-female ratio 21:10) participated in a 2-arm intervention trial from November 30 to December 19, 2020. The participants were instructed to indicate their momentary mood and physical symptoms (depressive mood, anxiety, stress, sleepiness, fatigue, and neck and shoulder stiffness) 5 times a day using a smartphone app. In addition, daily work performance was rated once a day after work. They were randomly assigned to either a feedback or control group, wherein they did or did not receive messages about their sleep status on the app every morning, respectively. All participants wore activity monitors on their nondominant wrists, through which objective sleep data were registered on the web on a server. On the basis of the estimated sleep data on the server, personalized sleep feedback messages were generated and sent to the participants in the feedback group using the app. These processes were fully automated.

**Results:**

Using hierarchical statistical models, we examined the differences in the statistical properties of sleep variables (sleep duration and midpoint of sleep) and daily work performance over the trial period. Group differences in the diurnal slopes for mood and physical symptoms were examined using a linear mixed effect model. We found a significant group difference among within-individual residuals at the midpoint of sleep (expected a posteriori for the difference: −15, 95% credible interval −26 to −4 min), suggesting more stable sleep timing in the feedback group. However, there were no significant group differences in daily work performance. We also found significant group differences in the diurnal slopes for sleepiness (*P*<.001), fatigue (*P*=.002), and neck and shoulder stiffness (*P*<.001), which was largely due to better scores in the feedback group at wake-up time relative to those in the control group.

**Conclusions:**

This is the first mobile health study to demonstrate that objective push-type sleep feedback improves sleep timing of and physical symptoms in healthy office workers. Future research should incorporate specific behavioral instructions intended to improve sleep habits and examine the effectiveness of these instructions.

## Introduction

### Development of Mobile Health Technologies

Recent developments in internet and communication technologies, mobile sensing devices, and the Internet of Things (IoT) have enabled the acquisition of longitudinal multidimensional information, including real-time physiological, behavioral, and environmental data. For example, consumer-grade wearable fitness trackers (eg, Fitbit, Garmin, and Jawbone) can record health-related information such as physical activity, sleep, and heart rate objectively and repeatedly.

By using smartphones and their SMS text messaging, it is possible to provide effective support at the optimal time to promote awareness of one’s health condition and improve compliance with intervention trials. Mobile devices can be useful for changing health-related behaviors as a part of daily living. Following these technological advancements and their applicability, medical and public health practices supported by mobile devices, such as mobile health (mHealth) [1,2], have been attracting attention in recent years. Since 2017, more than 325,000 mHealth apps have become available in commercial app stores, and the number of available apps continues to grow [3]. mHealth apps have been used in several intervention trials targeting physical activity [4-6], smoking [7,8], and suicidal ideation [9], and their usefulness and effectiveness have been proven.

### Health Effects of Habitual Sleep Behavior

Sleep is a significant aspect of recovery. In fact, adequate and good-quality sleep is associated with better physical and mental health [10-12] and improved daytime functioning, including decreased physical symptoms [13,14], better work performance [15], and improved quality of life [16].

Although research interest has focused on the importance of sleep duration and quality, recent studies indicate that sleep timing and stability of habitual sleep behavior also play important roles in health. In fact, delayed sleep timing is associated with obesity [17], congestive heart failure [18], poor glycemic control [19], and increased severity of depressive symptoms [20,21]. Additional studies have indicated that variability in day-to-day sleep behavior (ie, duration, timing, and quality), referred to as intraindividual variability [22], is linked to physiological dysfunction [23,24], adverse medical and mental health conditions [25], and poor psychological well-being [26]. Therefore, the multifaceted monitoring and regulation of habitual sleep behavior can contribute to the prevention and support for physical and mental health problems.

### Applications of Mobile and Wearable Technologies to Improve Sleep

Sleep data, along with other health-related behavioral and physiological data, can be gathered in real time using mobile devices. The widespread use of mobile or wearable sensing devices (eg, bed sensors, smartphone apps, and activity monitors) makes it easier and more commonplace to monitor sleep behavior in real time [27]. Although most consumer devices do not have Food and Drug Administration clearance as medical devices, they are expected to provide opportunities to track habitual sleep behavior longitudinally in large-scale populations [28]. For instance, Crowley et al [29] attempted to incorporate consumer wearable devices into health-promoting trials. They investigated the efficacy of these devices in improving physical activity and sleep among 565 employees over a 12-month period and found that sleep duration increased steadily throughout the study period.

In addition, mHealth apps for treating sleep disturbance have been developed rapidly [30,31], and more than 2000 mHealth apps targeting sleep are presently available in commercial app stores [32]. Pulantara et al [33,34] developed the interactive Resilience Enhancing Sleep Tactics app and examined its clinical feasibility as a treatment for sleep behavior. They reported that using the app improved insomnia severity and overall sleep quality, and the app was not inferior to traditional in-person sleep treatment. Furthermore, Hoersch et al [35] and Kuhn et al [36] conducted randomized controlled trials and reported that participants who received mHealth interventions had improved insomnia severity and sleep quality compared with waitlisted control participants.

### Remaining Issues

Despite the rapid growth of mHealth apps and mobile sensing technologies, recent reviews have indicated that scientific trials examining the usefulness of mHealth apps are limited [32], and further research is required to test whether objective data enhance sleep outcomes [37]. This research investigating the usefulness of mHealth apps in enhancing sleep has several limitations. First, most mHealth trials have not assessed sleep behavior objectively [31], and the feedback provided depended on self-report assessments by the participants. While some studies incorporated wearable devices into the trials [38,39], they used the measurements only to assess the efficacy of the trial but not to objectivize the feedback. Given the importance of self-management for habitual sleep behavior to prevent future health problems, it is beneficial to implement objective sleep feedback into the apps. Second, previous mHealth studies focused on improvement in limited aspects of habitual sleep behavior, such as sleep quality and sleep duration [31], and the dynamic aspects of sleep, including intraindividual variability in sleep measurements over the trial period, have tended to be ignored. To the best of our knowledge, only 1 mHealth study by Murawski et al [40] reported the dynamic aspects of sleep behavior and found that variability in sleep timing, as assessed by a self-report questionnaire, was improved after their intervention. Studying the dynamic features in sleep behavior may provide insight into the typical properties of sleep self-regulation processes when responding to feedback messages or other interventions. Finally, in view of the wide range of effects of sleep, including psychological well-being, the covariant relationships of sleep with daytime functions, such as mood, physical symptoms, and work performance, should be examined. The covariant relationships may contain important information about subordinate effects and facilitate a comprehensive understanding of sleep self-management.

### Objective

The objective of this study was to conduct an mHealth trial sending objective push-type sleep feedback to healthy participants using a smartphone app and a wearable activity monitor. Specifically, we examined whether sending daily sleep feedback messages changed sleep behavior and self-reported symptoms of the participants, particularly depressive mood, anxiety, stress, sleepiness, fatigue, and neck and shoulder stiffness. We used exploratory analysis of the statistical properties of objectively measured sleep variables and the characteristics of momentary symptoms recorded during the day using ecological momentary assessment (EMA).

## Methods

### Study Design

In this study, we conducted a 2-arm intervention trial by performing random convenience sampling of office workers at an insurance company and stratifying them into control and feedback groups. By comparing the groups in terms of the characteristics of habitual sleep behaviors (sleep duration and midpoint of sleep), momentary symptoms (depressive mood, anxiety, stress, sleepiness, fatigue, and neck and shoulder stiffness), and daily work performance, we examined the effects of personalized sleep feedback. To minimize the memory distortion caused by retrospective recall, momentary symptoms were recorded on a smartphone app in real time. Habitual sleep behaviors were measured objectively using a wearable device. Possible extraneous variables, including pretrial psychological symptoms, habitual sleep behaviors, and work performance, were assessed before the trial.

### EMA Method

We used the EMA method to acquire momentary mood and physical symptom data (ie, depressive mood, anxiety, stress, fatigue, sleepiness, and neck and shoulder stiffness) in real time. EMA is a method for recording participants’ behavior, psychological state, and physical symptoms in real time and at multiple time points, allowing the collection of self-report and objective data with reliability and ecological validity. Thus, EMA avoids potential distortions of retrospective recall in self-reported data [41,42].

### Health Care Internet of Things System

We developed a cloud-based health care Internet of Things (HIT) system that can continuously acquire health-related information, including momentary symptoms, biological signals, and surrounding environmental information, recorded as part of daily living. The HIT system consists of a cloud server and a smartphone app for data collection (HIT server and HIT app, respectively). The HIT app is equipped with an EMA and users can record their momentary symptoms in daily life (Multimedia Appendix 1). In addition, the HIT app can connect with various IoT devices, including a proprietary activity monitor (Sciencenet device, Sciencenet Inc) used in this study, using Bluetooth Low Energy (BLE). Data are transferred from the IoT devices to the HIT server. The app is compatible with both Android and iOS operating systems. The HIT server can store, integrate, and manage data uploaded from the app and send personalized messages (push-type feedback messages) to the HIT app users. HIT systems have been used to assess self-reported symptoms in real time [43].

### Participants

A convenience sample of 31 office workers working at an insurance company participated in this study. The mean age of the participants was 42.3 (SD 7.9) years, and the male-to-female ratio was 21:10. All participants were working from home during the trial period at the request of their employer to prevent the spread of COVID-19.

Participants were randomly assigned to a control or feedback group using the “sample” function in the R statistical software (version 4.0.2; R Foundation for Statistical Computing) so that the ratio of the sample size was 1:1. No stratification by age or sex was observed. Coauthor KS conducted this randomization, independent of the primary researcher HT. Although author HT was also informed of who was assigned to which group after the random assignment, he was not allowed to contact the participants during the trial period.

During the trial period, the participants in the feedback group received personalized messages regarding their current sleep status every morning, whereas the participants in the control group did not receive any messages. The control group consisted of 16 participants, including 9 males and 7 females, with a mean age of 44.1 (SD 8.3) years. The feedback group consisted of 15 participants, including 12 males and 3 females, with a mean age of 40.5 (SD 7.2) years.

### Instruments

#### Baseline Questionnaire

Before the trial, the participants completed a baseline questionnaire, including their demographic information (age, sex, and BMI), psychological symptoms (depressive and anxiety symptoms), habitual sleep behaviors (habitual sleep duration and self-reported sleep quality), and self-reported work performance. Items included in the baseline questionnaire are listed in subsequent sections.

#### Psychological Symptoms

Depressive symptoms were assessed using the Japanese version of the Beck Depression Inventory second edition (BDI-II) [44,45]. The BDI-II is a 21-item self-report inventory for measuring the presence and severity of depression (score range 0-63). A high level of internal consistency (Cronbach α=.87) and item homogeneity have been confirmed for the Japanese version of the BDI-II [45]. The BDI-II classifies individuals into 4 categories based on an overall score: minimal or no depression, 0 to 13; mild depression, 14 to 19; moderate depression, 20 to 28; and severe depression, 29 to 63. A score of ≥14 points was used as the clinical cutoff point for depression.

Anxiety symptoms were assessed using the Japanese version of the State-Trait Anxiety Inventory (STAI) Form Y [46]. The STAI is a standardized self-report inventory for measuring state and trait anxiety with 20 items (STAI Y-1 and STAI Y-2, respectively). The STAI Y-1 measures the intensity of the anxiety felt by an individual in the present, whereas the STAI Y-2 measures how often an individual feels anxious. Scores range from 20 to 80 for each subscale, with higher scores indicating higher levels of anxiety.

#### Habitual Sleep Behaviors

Habitual sleep duration on workdays and free days (SL*_w_* and SL*_f_*, respectively) was assessed using a single question for each (“How long do you sleep on weekdays?” and “How long do you sleep if tomorrow is a holiday?”). These measurements were used to compute an index representing participants’ sleep status during the trial period. Sleep quality was assessed using the Pittsburgh Sleep Quality Index (PSQI). The PSQI is a self-report inventory used to assess sleep quality over the preceding month [47,48]. The PSQI consists of 19 items on self-reported sleep quality, sleep latency, sleep duration, habitual sleep efficiency, sleep disturbances, use of sleeping medication, and daytime dysfunction. Scores range from 0 to 21, with a higher total score indicating poorer sleep quality. The strong reliability and validity of this questionnaire have been confirmed in a recent meta-analysis [49].

#### Self-reported Work Performance

Self-reported work performance was measured using the World Health Organization Health and Work Performance Questionnaire (HPQ) [50,51]. The HPQ asks participants to rate their overall work performance over the preceding 4 weeks on a self-anchoring scale from 0 to 10: “On a scale of 0 to 10, how would you rate your usual work performance over the past four weeks?” The score was converted to a 100-point scale by multiplying the raw score by 10, with a higher score indicating better work performance.

### EMA Questionnaire

The participants answered an EMA questionnaire 5 times per day using the HIT app. The EMA included the following measurements:

1. Depressive mood and anxiety were scored using the Depression and Anxiety Mood Scale [52]. This scale comprises the following 9 adjectives representing mood states: “vigorous,” “gloomy,” “concerned,” “happy,” “unpleasant,” “anxious,” “cheerful,” “depressed,” and “worried.” On the basis of the 9 items, anxious (the sum of “concerned,” “anxious,” and “worried” scores), positive (the sum of “vigorous,” “happy,” and “cheerful” scores), and negative (the sum of “gloomy,” “unpleasant,” and “depressed” scores) moods were calculated. Depressive mood scores were obtained by combining the last 2 mood scores as follows: (300-positive mood score) +negative mood score. The resulting depressive mood scores were rescaled to range from 0 to 100.
2. Physical symptoms, including stress, sleepiness, fatigue, and neck and shoulder stiffness were rated according to the participant’s response to being asked if they felt “stressed,” “sleepy,” or “fatigued” and “if their neck and shoulders were stiff.”
3. Daily work performance was rated after work in response to the question, “How would you rate your work performance of today?”

These measurements were rated using a visual analog scale from 0 to 100 displayed on the screen. All scores were transferred to the HIT server immediately after the completion of each EMA questionnaire.

### Sleep Monitoring

The participants were instructed to wear a wristband-type activity monitor on their nondominant wrist during the trial period, except while bathing, showering, performing rigorous exercise, or any other activities likely to damage the device. The device is equipped with triaxial piezoelectric accelerometers capable of detecting small changes in bodily acceleration (≥0.01 G/rad/s). We confirmed that the device performs at a level equivalent to research-grade actigraphy (Ambulatory Monitors Inc), which is widely used in clinical settings. Results of the comparative analysis are presented in Multimedia Appendix 2.

The device was configured to transfer physical activity data to the HIT server using the HIT app whenever a participant launched the app. We used zero-crossing counts, which counts the number of times per epoch that the acceleration signal level crosses 0 [53], accumulated per minute, to compute objective sleep variables.

To estimate sleep variables, we adopted the Cole-Kripke algorithm with Webster’s rescoring rules [54,55] for zero-crossing count data to identify whether the recorded 1-minute epoch was sleep or wake. Next, we introduced the sleep probability function (SPF) θ*(t)* as follows:



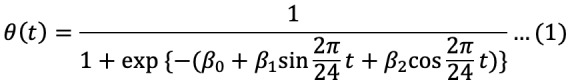



where *t* denotes the clock time converted to a numerical value (eg, 3 AM is transformed to 3.00, and 6:30 PM is converted to 18.50). Thus, the SPF is a value ranging from 0 to 1 and represents circadian oscillations.

Then, we estimated the effective SPF parameters (β_0_, β_1_, and β_2_) to fit the actual Cole-Kripke identification using a Bernoulli logistic regression model.


CK*(t)~bernoulli(θ(t))...*
**(2)**

where CK(*t*) represents the result of Cole-Kripke identification when the clock time is *t*; CK(*t*) = 0 and CK(*t*) = 1 denote that the epoch at time *t* was labeled as wake and sleep, respectively.

Finally, we estimated the square waveform function θ’(*t*) from θ(*t*) by introducing onset and offset (*t_on_* and *t_off_*, respectively).



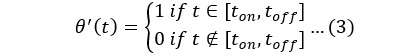



Effective *t_on_* and *t_off_* were computed to maximize the *R*^2^ value between θ’(t) and CK(t) using the Nelder-Mead method. We assumed that *t_on_* and *t_off_* represent bedtime and wake-up time, respectively; thus, sleep duration and midpoint of sleep were determined using their interval and midpoint. We confirmed that the algorithm was performed at a level equivalent to Action-W version 2 software (AW2 software, Ambulatory Monitors Inc), which was used to analyze the research-grade actigraphy data (Multimedia Appendix 2).

The average sleep duration per day was calculated based on the habitual sleep duration on workdays and free days (*S_w_* and *S_f_*, respectively) in the baseline questionnaire using the following equation:



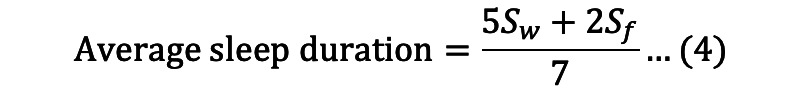



which represents the expected sleep duration per day because the participants worked 5 days per week. Sleep debt and cumulative sleep debt were defined as follows:


sleep debt = Average sleep duration − Estimated sleep duration...**(5)**

cumulative sleep debt = Σsleep debt...**(6)**

Thus, sleep debt represents sleep insufficiency per day, and cumulative sleep debt represents its cumulative value over time. During the trial period, these values were automatically sent to the participants in the feedback group using the HIT app.

### Data Collection Protocol

Trained researchers provided participants with a comprehensive explanation of the purpose and potential risks of the study. Subsequently, they signed an informed consent form and completed the baseline questionnaire. In addition, they received a sleep hygiene guide that listed daytime activities to improve their habitual sleep behaviors or health conditions (Multimedia Appendix 3). They were then asked to install the HIT app on their smartphones and wear an activity monitor on their nondominant wrist. All participants were instructed on the use of the app and the activity monitor. Using these instruments, we repeatedly measured their momentary symptoms and physical activity data in real time. The setup and operating procedures were presented on the web as much as possible using a communication service and videoconferencing system.

**Figure 1:**
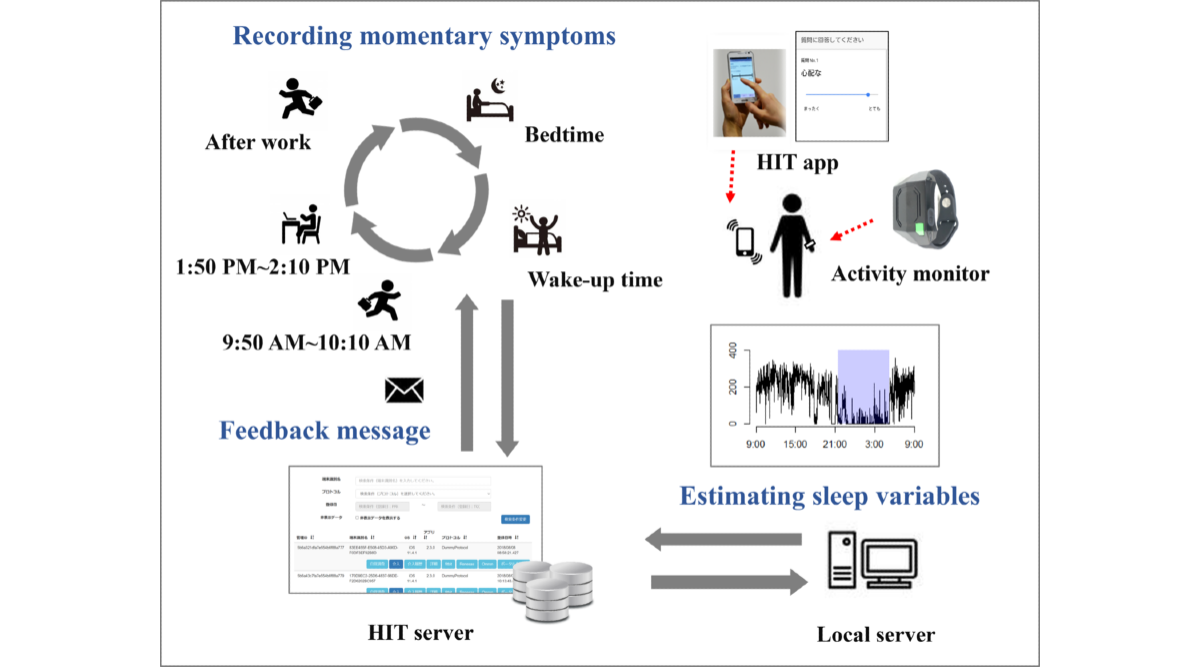
Overview of the trial. HIT: health care Internet of Things.

An overview of the trial is shown in Figure 1. This trial was carried out for almost 3 weeks (from November 30 to December 19, 2020). During the trial period, the participants were asked to complete the EMA questionnaires (see the section EMA Questionnaire) at randomly selected times within +10 minutes to −10 minutes of predetermined times (10 AM and 2 PM). In addition, they were asked to complete the EMA when they woke up, finished their work, and went to bed (wake-up time, after work, and bedtime, respectively).

At 9 AM every day, the physical activity data on the HIT server were collated and analyzed to estimate sleep duration, sleep debt, and cumulative sleep debt using a local data analysis server. On the basis of the estimated sleep data, personalized sleep feedback messages were generated and sent to participants in the feedback group. They were informed about their sleep status (estimated sleep debt and cumulative sleep debt) and requested to plan and adjust their daytime activities with reference to guidelines for reducing their sleep debt. The message read as follows: “You accumulated XX minutes of sleep debt yesterday. Your current overall debt is XX minutes. Sleep debt has adverse effects on physical and mental health. Adjust your daytime behavior to cancel your debt.”

If sufficient physical activity data (<720 records/day) had not been uploaded to the server by 9 AM, an alternative message was sent to the participant requesting them to confirm the BLE pairing of the activity monitor with their app: “It seems that your data have not been uploaded successfully. We will analyze the data again at 1 PM. Please check the BLE connection of your HIT app before then.” The same computation process was executed at 1 PM for the relevant participants. These processes were executed by the local servers and were fully automated.

After the trial period, the participants rated their overall work performance in the preceding 2 weeks on a self-anchoring scale from 1 to 10. The question was as follows: “On a scale from 0 to 10, how would you rate your work performance over the past two weeks?” While the HPQ was originally developed to evaluate work performance over the past month, the scale used in this study was modified to evaluate performance over the past 2 weeks, corresponding to the survey period.

### Statistical Analysis

Sleep variables, including sleep duration and midpoint of sleep, and daily work performance were recorded once a day, while mood and physical symptoms were recorded several times a day using smartphone-based EMA. Owing to this difference in the frequency of data recording, we used different models to analyze the group differences in the obtained data.

For the sleep variables and daily work performance, we constructed a hierarchical Bayesian model to capture the daily trend, baseline level, and within-individual stability, as follows:


*y_ijk_ = β_ij_*
^0^
*+ β_i_*
^1^
*Day_ijk_ + e_ijk_*

*β_ij_*
^0^
*= β_i_*
^0^
*+ r_ij_*
^0^

*e_ijk_ ~ N*(0*, σ_i_*^y^)

*r_ij_*^0^*~ N*(0*,σ*^0^)

where *y_ijk_* indicates the dependent variable (sleep duration, midpoint of sleep, or daily work performance) at the *k*-th recording for the *j*-th participant in the *i*-th group; *Day_ijk_* indicates the day on which the corresponding dependent variable was measured to record the trend (improving or worsening); *β_ij_*^0^ is the intercept of the *j*-th participant in the *i*-th group; *β_i_*^1^ is the slope for the day of the *i*-th group; the random terms *r_ij_*^0^ are the between-individual residuals; and *e_ijk_* are the within-individual residuals. In particular, the variance component σ_i_^y^ can be interpreted as a measurement of how stable or fluctuating the sleep variables within an individual are at the group level. Therefore, σ_i_^y^ was assumed to be affected by whether the intervention was provided and estimated for each group. All random terms were assumed to follow a normal distribution.

For EMA-recorded mood and physical symptoms, we used linear mixed effect models to examine their diurnal slopes and group differences.


*y_ijk_ = β_j_*
^0^
*+ β*
^1^
*Time_ijk_ + β*
^2^
*Group_ijk_ + β*
^3^
Time_ijk_ * Group_ijk_ + e_ijk_

*β_j_*
^0^
*= β*
^0^
*+ r_j_*
^0^

*e_ijk_ ~ N*(0, *σ*^y^)

*r_j_*^0^*~ N*(0,*σ*^0^)

where *y_ijk_* indicates the moods or physical symptoms at the *k*-th recording for the *j*-th participant in the *i*-th group; *Time_ijk_* indicates the categorical variable representing the timing when the corresponding dependent variables were recorded; thus, Time*_ijk_* = 0 and Time*_ijk_* = 1 denote that the dependent variables were recorded at wake-up time and bedtime, respectively; *Group_ijk_* indicates the categorical variable representing the group in which the *j*-th participant was classed; thus, Group*_ijk_* = 0 and Group*_ijk_* = 1 denote that the dependent variable was obtained from the participant classed as the control and feedback groups, respectively; *β_j_* ^0^ is the intercept of the *j*-th participant; *β*^1^, *β*^2^, and *β*^3^ are the coefficients for *Time_ijk_*, *Group_ijk_*, and their interaction term, respectively; the random terms *r_j_*^0^ are the between-individual residuals and *e_ijk_* are the within-individual residuals. All random terms were assumed to follow a normal distribution. When a significant interaction effect was observed, we performed multiple comparison tests with Tukey correction.

In addition, we performed two-tailed Welch *t* test and Fisher exact test for the baseline data to confirm that there were no significant group differences. As the HPQ scores were recorded in the baseline and follow-up questionnaires, we performed 2-way repeated measures ANOVA to examine the main effects of group (control vs feedback) and time (pre- vs postintervention) and their interaction effect. For the cumulative sleep debt, we performed the Welch 2-tailed *t* test and Levene test for equality of variances for the final observation data per participant to examine the group differences in terms of mean and variance.

All analyses were performed using R statistical software (version 4.0.2). In particular, the parameters of the statistical models were computed using the rstan [56] and lmerTest [57] packages. The emmeans package (also known as the lsmeans package) [58] was used for multiple comparison tests with Tukey correction. Statistical significance was defined as when the 95% credible interval (CI) did not include the null value or when a *P* value <.05.

### Ethics Approval

The Ethics Committee of the University of Tokyo approved this study and the informed consent form (approval number 20-20).

## Results

### Demographic Characteristics

**Figure 2:**
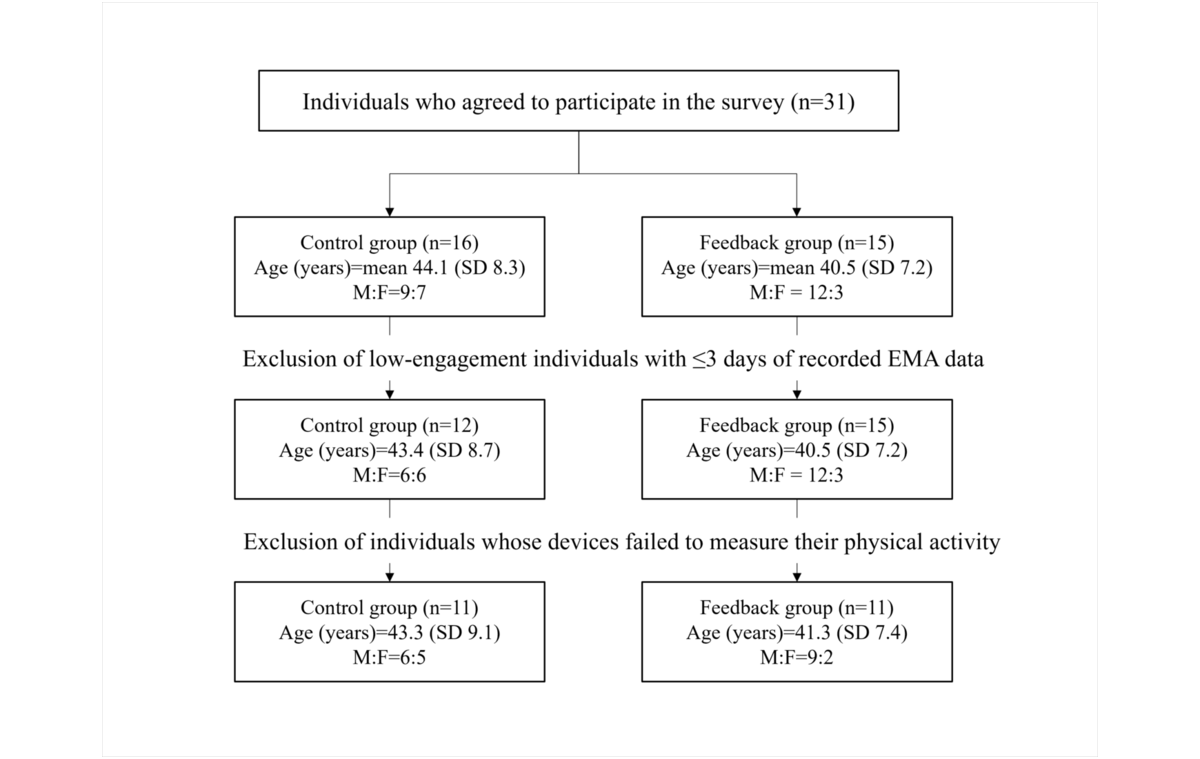
Flowchart of participant data selection for analyses. EMA: ecological momentary assessment; F: female; M: male.

In total, 31 individuals agreed to participate in the study. However, the data of 4 participants were excluded from the statistical analyses because of their low response rates for wake-up time and bedtime on the EMA questionnaire (ie, <3 days; Figure 2). The demographic characteristics of the participants, including the control group (n=12) and the feedback group (n=15), are presented in Table 1. The mean age of the participants was 41.8 (SD 7.9) years, and 33% (9/27) were females. The Welch 2-tailed *t* test and Fisher exact test showed that there were no significant differences in demographic characteristics between the groups. The total number of EMA records was 1839, and the overall response rate was 64.88% (1839/2835).

**Table 1:** Baseline demographic characteristics of participants by group.

	Overall (N=27)	Control group (n=12)	Feedback group (n=15)	*P* value
Age (years), mean (SD)	41.78 (7.90)	43.42 (8.72)	40.47 (7.21)	.35^a^
Female, n (%)	9 (33)	6 (50)	3 (20)	.22^b^
BMI (kg/m^2^), mean (SD)	21.65 (2.60)	21.18 (2.54)	22.03 (2.67)	.40^a^
BDI-II^c^, mean (SD)	6.51 (5.13)	6.92 (4.94)	6.20 (5.42)	.72^a^
BDI-II >13, n (%)	4 (15)	2 (17)	2 (13)	.99^b^
STAI Y-1^d^, mean (SD)	39.78 (11.05)	38.75 (11.31)	40.60 (11.17)	.67^a^
STAI Y-2^e^, mean (SD)	40.74 (10.28)	38.67 (8.50)	41.20 (11.69)	.52^a^
PSQI^f^, mean (SD)	4.73 (2.16)	4.75 (2.05)	4.71 (2.33)	.97^a^
S_w_^g^, mean (SD)	6 h 18 min (1 h 4 min)	6 h 20 min (1 h 18 min)	6 h 16 min (53 min)	.88^a^
S_f_^h^, mean (SD)	6 h 49 min (1 h 23 min)	6 h 35 min (1 h 23 min)	7 h 00 min (1 h 25 min)	.45^a^

^a^Welch *t* test.

^b^Fisher exact test.

^c^BDI-II: Beck Depression Inventory second edition.

^d^STAI Y-1: State Anxiety Scale.

^e^STAI Y-2: Trait Anxiety Scale.

^f^PSQI: Pittsburgh Sleep Quality Index.

^g^S_w_: sleep duration on work days.

^h^S_f_: sleep duration on free days.

### Statistical Properties of Sleep Variables

We examined group differences in the sleep variables such as cumulative sleep debt, sleep duration, and midpoint of sleep during the trial period. The data of 5 of the 27 (18%) participants were excluded from the analyses because of physical activity measurement failures (Figure 2). Thus, the data from 11 participants in the control group (mean age 43.3, SD 9.1 years; 6 males and 5 females) and 11 participants in the feedback group (mean age 41.3, SD 7.41 years; 9 males and 2 females) were analyzed.

**Figure 3:**
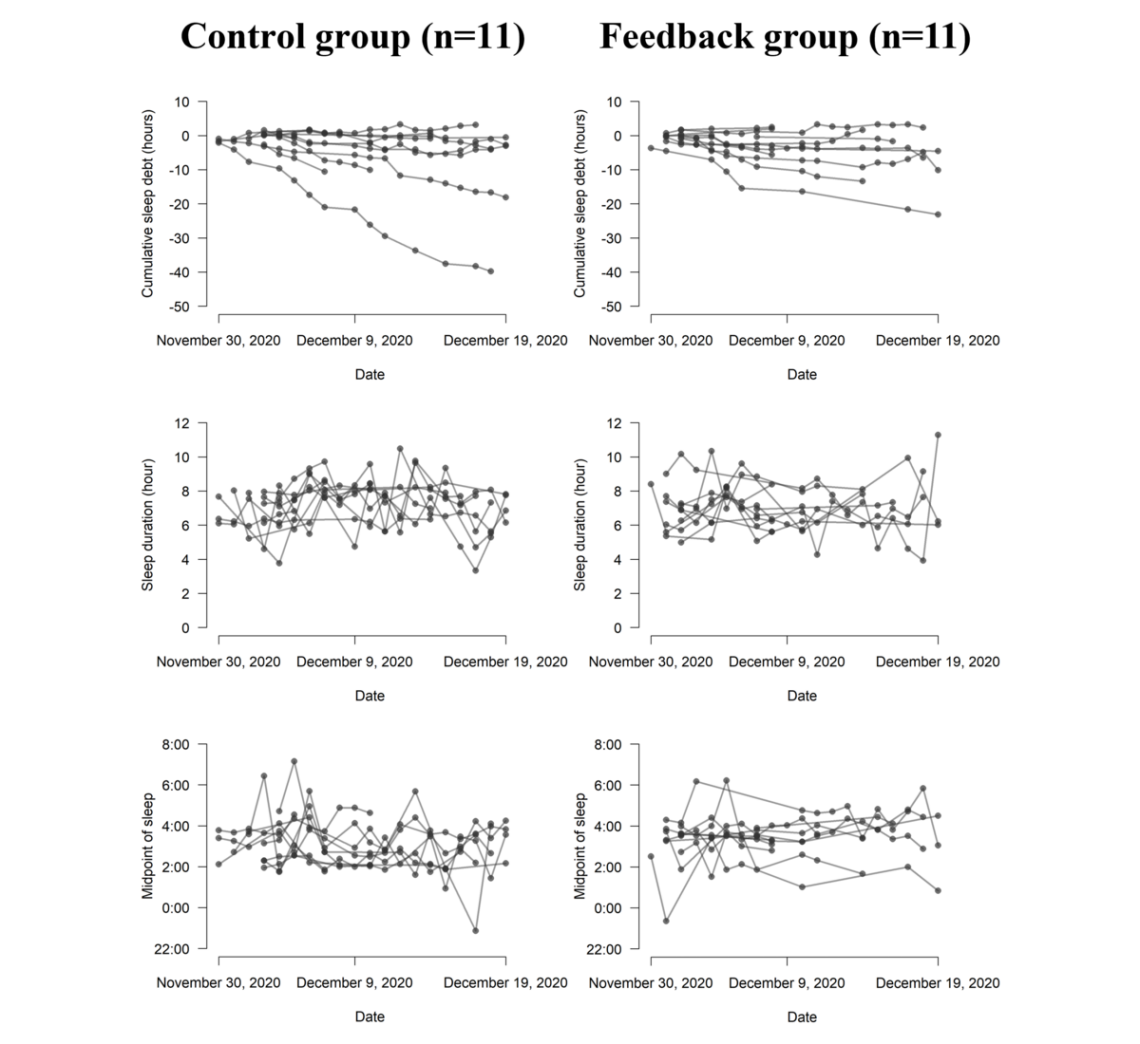
Spaghetti plots of the estimated cumulative sleep debt (top panels), sleep duration (middle panels), and midpoint of sleep (bottom panels) per participant across the trial period. The left and right panels indicate the time series of the sleep variables for the control group and the feedback group, respectively.

Figure 3 presents the spaghetti plots of the computed sleep variables for all participants during the trial period. The Welch *t* test and Levene test for equality of variances indicated that there was no significant group difference in cumulative sleep debt per participant, t_17.32_=0.64, *P*=.53; *F*_1,20_=0.15, *P*=.70.

We subsequently examined the group differences in the daily trend, baseline level, and within-individual residuals for sleep duration and midpoint of sleep using the hierarchical Bayesian model. The within-individual residuals for the midpoint of sleep in the feedback group were significantly smaller than those in the control group (expected a posteriori for the difference: −15, 95% CI −26 to −4 min; Table 2). This was also the case for the within-individual residuals for bedtime (−18, 95% CI−31 to −4 min; Multimedia Appendix 4). Both groups showed no significant slope of the day in terms of sleep duration (control group: 0 95% CI −3 to 3 min; feedback group: 1, 95% CI −3 to 1 min) or midpoint of sleep (control group: −1, 95% CI −4 to 1 min; feedback group: 1, 95% CI −1 to 3 min).

**Table 2:** Results of the hierarchical Bayesian model for sleep duration and midpoint of sleep.

		Control group			Feedback group			Difference	
		EAP^a^ (SD)^b^	95% CI^c^	EAP (SD)		95% CI	EAP (SD)		95% CI
**Sleep duration^d^**									
	Intercept	7 h 33 min (26 min)	6 h 42 min to 8 h 23 min	7 h 31 min (33 min)		6 h 27 min to 8 h 36 min	−1 min (31 min)		−1 h 2 min to 58 min
	Day	0 min (1 min)	−3 min to 3 min	1 min (1 min)		−3 min to 1 min	−1 min (2 min)		−4 min to 3 min
	σ^0e^	48 min (11 min)	29 min to 1 h 14 min	48 min (11 min)		29 min to 1 h 14 min	N/A^f^		N/A
	σ^y^	1 h 13 min (5 min)	1 h 4 min to 1 h 23 min	1 h 13 min (6 min)		1 h 3 min to 1 h 26 min	0 min (8 min)		−14 min to 16 min
**Midpoint of sleep^d^**									
	Intercept	3:30 (21 min)	2:48 to 4:12	3:34 (26 min)		2:44 to 4:26	4 min (24 min)		−42 min to 52 min
	Day	−1 min (1 min)	−4 min to 1 min	1 min (1 min)		−1 min to 3 min	2 min (1 min)		0 min to 5 min
	σ^0^	41 min (9 min)	27 min to 1 h 1 min	41 min (9 min)		27 min to 1 h 1 min	N/A		N/A
	σ^y^	1 h 1 min (4 min)	53 min to 1 h 9 min	46 min (4 min)		39 min to 54 min	−*15 min* (*5 min*)^g^		−*26 min to −4 min*

^a^EAP: expected a posteriori (expected value of the posterior distribution).

^b^SD of the posterior distribution.

^c^CI: credible interval.

^d^The models were run after controlling for age and sex.

^e^The difference in the interindividual variability for the intercept (σ^0^) was not computed because σ^0^ was assumed to be equal between groups.

^f^N/A: not applicable.

^g^Italicized values denote statistically significant group effects, at a 95% CI.

### Self-reported Work Performance Assessment

**Figure 4:**
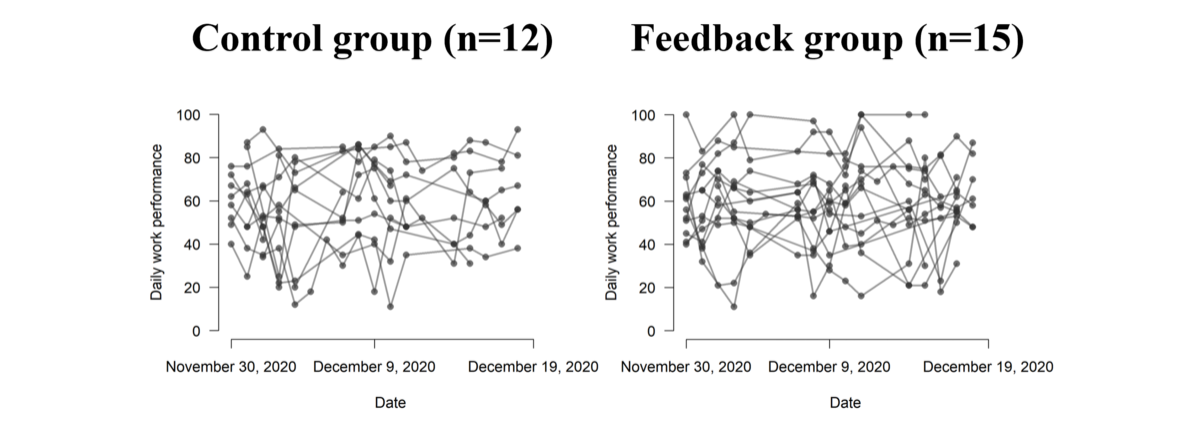
Spaghetti plots of the daily work performance recorded using ecological momentary assessment per participant across the trial period. The left and right panels indicate the time series of the work performance for the control group and the feedback group, respectively.

We examined the group differences in daily work performance during the trial period using 307 EMA records obtained from 27 participants (Figure 4). We examined the statistical properties of daily work performance by using the statistical model used to analyze the sleep variables. However, there were no significant differences between the groups (Table 3). When comparing the HPQ score before and after the intervention using a 2-way repeated ANOVA, the main effects of group, *F*_1,25_=0.39, *P*=.54, and time, *F*_1,25_=0.01, *P*=.94, and their interaction, *F*_1,25_=0.05, *P*=.83, were not significant.

**Table 3:** Results of the hierarchical Bayesian model for daily work performance.

			Control				Feedback				Difference		
			EAP^a^ (SD)^b^		95% CI^c^		EAP (SD)		95% CI		EAP (SD)		95% CI
**Daily work performance^d^**													
	Intercept	57.59 (6.67)		44.60 to 70.54		61.45 (8.77)		44.24 to 78.82		3.85 (8.01)		−11.92 to 19.84	
	Day	0.32 (0.22)		−0.11 to 0.75		0.08 (0.17)		−0.26 to 0.41		−0.24 (0.27)		−0.77 to 0.29	
	σ^0e^	17.06 (2.89)		12.38 to 23.54		17.06 (2.89)		12.38 to 23.54		N/A^f^		N/A	
	σ^y^	13.28 (0.88)		11.68 to 15.14		12.62 (0.72)		11.31 to 14.12		−0.66 (1.14)		−2.94 to 1.50	

^a^EAP: expected a posteriori.

^b^SD of the posterior distribution.

^c^CI: credible interval.

^d^The models were run after controlling for age and sex.

^e^The difference in the interindividual variability for the intercept (σ^0^) was not computed because σ^0^ was assumed to be equal between groups.

^f^N/A: not applicable.

### Diurnal Slopes for EMA Scores

**Figure 5:**
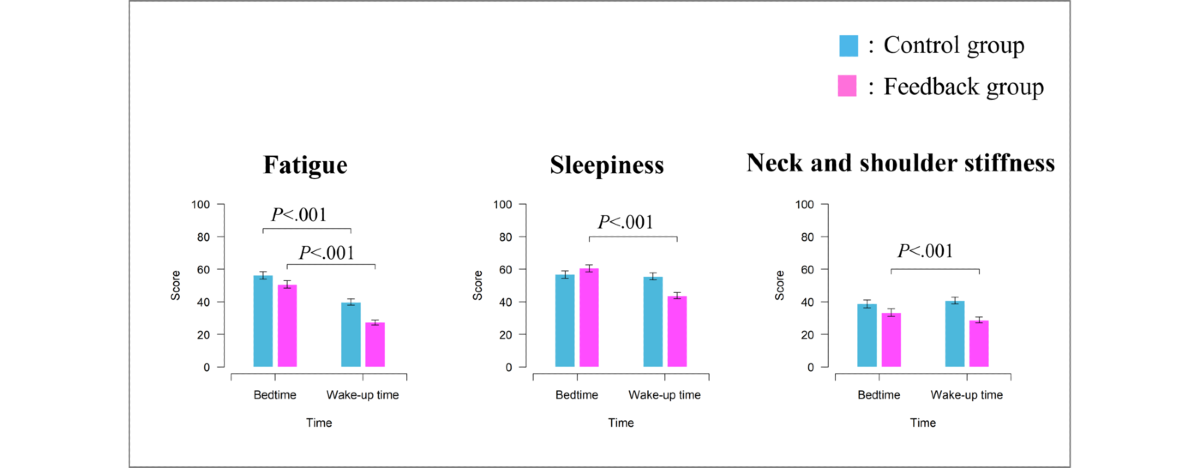
Average ecological momentary assessment scores for fatigue, sleepiness, and neck and shoulder stiffness at bedtime and wake-up time in the control group and the feedback group. The error bars indicate the SE per group.

The recorded EMA scores of 27 participants (760 records) were used to examine the diurnal slopes in momentary mood and physical symptoms (Figure 2). The linear mixed effect model results showed significant interaction effects between group and time in physical symptoms (fatigue, *P*=.002; sleepiness, *P*<.001; and neck and shoulder stiffness, *P*<.001; Table 4). In addition, a multiple comparison test with Tukey correction showed that the EMA scores of physical symptoms at wake-up time were significantly lower than those at bedtime in the feedback group (fatigue, sleepiness, and neck and shoulder stiffness, *P*<.001). In the control group, a significant difference between wake-up time and bedtime was observed only for fatigue (*P*<.001; Figure 5).

**Table 4:** Results of the linear mixed effect model for mood and physical symptoms.

Mood and physical symptoms^a^			Coefficient (SE)		Df^b^		*P* value
**Depressive mood**							
	Intercept^c^	41.27 (5.56)		23.28		<.001	
	Group	−9.87 (6.61)		23.42		.15	
	Time	−0.35 (1.10)		731.32		.75	
	Group×Time	0.74 (1.51)		731.6		.63	
**Anxiety**							
	Intercept^c^	35.75 (7.42)		23.31		<.001	
	Group	−16.89 (8.82)		23.46		.07	
	Time	−1.29 (1.56)		731.36		.41	
	Group×Time	3.03 (2.14)		731.66		.16	
**Stress**							
	Intercept	47.07 (8.02)		23.42		<.001	
	Group	−18.66 (9.54)		23.6		.06	
	Time	2.49 (1.80)		731.46		.17	
	Group×Time	0.46 (2.47)		731.82		.85	
**Fatigue**							
	Intercept	35.22 (7.24)		23.72		*<.001* ^d^	
	Group	−16.47 (8.62)		24.03		*.07*	
	Time	16.07 (2.14)		731.78		*<.001*	
	Group×Time	9.31 (2.96)		732.39		*.002*	
**Sleepiness**							
	Intercept	55.75 (7.20)		24.08		*<.001*	
	Group	−9.81 (8.58)		24.51		*.26*	
	Time	1.27 (2.49)		732.14		*.61*	
	Group×Time	17.70 (3.42)		732.96		*<.001*	
**Neck and shoulder stiffness**							
	Intercept	43.33 (9.59)		23.27		*<.001*	
	Group	−12.99 (11.40)		23.37		*.27*	
	Time	−2.51 (1.64)		731.3		*.13*	
	Group×Time	8.46 (2.26)		731.51		*<.001*	

^a^The df values correspond to the denominator df in ANOVA model.

^b^The effects of group (control vs feedback group) and time (wake-up time vs bedtime) were assumed to be fixed effects, and those of individuals were assumed to be random effects.

^c^The control group and wake-up time were used as the reference categories for each variable; thus, the intercept indicates the expected EMA score of moods or physical symptoms for the control group during the wake-up time.

^d^Italicized values denote statistically significant interaction effects. All models were run by controlling for age and sex.

## Discussion

### Principal Findings

We explored the effects of personalized feedback messages regarding the current sleep status on habitual sleep behavior and momentary mood and physical symptoms in Japanese office workers, using a unique cloud-based HIT system that included a web-based wearable activity monitor and a smartphone app. Specifically, we focused on group differences in the statistical properties of sleep variables and within-day momentary symptoms during the trial period. We found that the within-individual residuals for sleep timing were significantly smaller in the feedback group than in the control group. In addition, the diurnal slopes for physical symptoms (sleepiness, fatigue, and neck and shoulder stiffness) differed significantly between the feedback and control groups, largely because of better physical symptom scores in the feedback group at wake-up time. This is the first mHealth study to implement push-type sleep feedback based on objective measurements and to demonstrate improved sleep status and momentary symptoms associated with receiving the feedback message. The findings in this study suggest that objective push-type feedback messages may promote sleep self-management and solve habitual sleep behavior problems, despite the minor inconvenience.

Several mHealth apps have been developed for treating sleep disturbances such as insomnia [30,31], but the objective sleep feedback function has not been implemented in these apps. While users of consumer-grade wearable devices can take advantage of objective sleep measurements, these are regarded as pull-type interventions, as these devices require the user to actively access an app to receive feedback, which can sometimes be burdensome. Connecting mHealth apps with wearable sensing devices and sending objective push-type sleep feedback may be a feature to consider when developing or updating mHealth apps that target sleep disturbance.

### Comparison With Previous Work

The within-individual residuals in sleep variables examined in this study can represent how sleep behavior varies across days, which is commonly referred to as intraindividual variability [22]. Recently, the stability of habitual sleep behavior has been considered a critical factor for physical and mental health, as previous studies have indicated that greater intraindividual variability in sleep behavior is associated with worse medical health conditions [25] and poorer psychological well-being [26]. In our study, participants may have attempted to improve their sleep habits by adopting strategies to stabilize their sleep timing (specifically their bedtime); for example, by not staying up late excessively. This inference is supported by evidence of a significant group difference in the within-individual residuals at bedtime but not in wake-up time. Similar results were reported by Murawski et al [40] who found improvement in the variability of sleep timing after the 3-month intervention. Given the fact that our survey period was relatively short, it is speculated that the improvement in sleep variability is an initial change caused by improved awareness of habitual sleep behaviors. Especially in modern industrial societies, the sleep-wake cycle adhering to social schedules, rather than endogenous circadian rhythms, leads to exposure to bright light at significantly different times from the natural environment. This causes disturbances in sleep and circadian rhythms, such as circadian misalignment [59] and social jet lag [60,61], which are linked to future health problems. Thus, the findings of this study suggest that sending personalized sleep feedback messages may potentially contribute to the primary prevention of physical and mental health problems as well as the improvement of the sleep-wake cycle.

Improvements in physical symptoms and stabilization of sleep timing were simultaneously observed in the feedback group, suggesting the covariant relationship between them. Indeed, previous studies have indicated that individuals with greater intraindividual variability in sleep timing and sleep duration show more dysregulated biomarkers related to endogenous circadian rhythm [23] and inflammatory functions [24], which can influence diurnal symptoms including sleepiness and fatigue. Therefore, improved physical symptoms at wake-up time in the feedback group may have been caused by stabilized sleep timing, mediated by regulated physiological systems. Thus, it is possible that improved physical symptoms were observed as a short-term effect or proximal outcome of the interventions that stabilized sleep timing and, by extension, functioned as an incentive to improve sleep habits and adjust daytime activities. However, inconsistent results have been reported in previous studies investigating the relationship between sleep and physical symptoms. In a cross-sectional study, there was no relationship between sleep variability and daytime fatigue and sleepiness [62], whereas daytime sleepiness was reduced among university students who were instructed to stabilize their sleep-wake schedule experimentally [63]. These mixed results may be due to the use of survey designs that evaluate symptoms at a single time point. In contrast, we measured physical symptoms several times per day using the EMA technique, with finer temporal resolution than in previous studies, and with ecological validity, resulting in the discovery of improved physical symptoms at wake-up time in the feedback group. When acquiring physical symptom data, the timing of the measurements can also be an important factor.

A previous study demonstrated that the use of wearable devices improved sleep duration in a healthy population [29]. However, the hierarchical Bayesian model demonstrated that the slope of day for sleep duration and midpoint of sleep were not statistically significant; thus, we did not find an improvement in sleep duration or midpoint of sleep by using only sleep feedback. This discrepancy may be due to the prompts of feedback messages. During the survey period, participants’ sleep debt did not accumulate much, and they seemed to receive messages indicating that their sleep status was better. Under such circumstances, they might have attempted to maintain their sleep status by stabilizing their sleep-wake cycle rather than to improve their sleep duration or sleep timing. In future studies, it will be necessary to provide further support for prolonging sleep duration and advancing sleep timing, in addition to an objective sleep feedback message. Improvements in sleep duration and timing can also be beneficial to daytime functions, such as momentary moods and work performance, which were not improved in this study.

### Future Directions

The HIT app is primarily designed for collecting multidimensional data in daily life, unlike mHealth apps developed for treating sleep disturbance. Therefore, in the HIT app, the functions useful for improving sleep disturbances are limited to objective feedback messages, while other apps offer several support functions, such as psychoeducation, sleep hygiene, and data visualization [30]. It is possible to improve sleep duration and timing by incorporating additional intervention options into the HIT app. Behavioral instructions to build sufficient sleep pressure at night are an example of additional support. In the treatment of patients with insomnia, sleep restriction therapy, which induces mild sleep deprivation to build homeostatic sleep pressure, is used, and existing mHealth studies incorporating sleep restriction therapy demonstrate a significant improvement in insomnia severity and sleep efficacy [35,64,65]. Interventions that build sufficient homeostatic sleep pressure as part of daily living, for example, exercising in the evening and avoiding long naps, may improve sleep duration and daytime functions, including work performance. Other interventions include behavioral coordination that works on the endogenous circadian rhythm; for example, adjusting the timing of food intake and avoiding exposure to bright light before bedtime. Such expansions can facilitate the control of habitual sleep timing and enhance the applicability of an IoT system with mobile devices for the treatment of various sleep disorders. Measuring, integrating, and using multidimensional information, including environmental and behavioral data, requires additional research. Simultaneously, integrative health care information systems, such as an HIT system, may provide a solution and expand intervention options, facilitating the verification of their effectiveness.

### Limitations

This study had several limitations. First, the small number of participants were recruited from a life insurance company in Japan, and the social schedules of participants appeared similar, limiting the generalizability of the results. Indeed, work-related factors, including occupation, job stress, work hours, shift work, and physically demanding work, are associated with habitual sleep duration and sleep quality [66-68], suggesting that sleep habits may differ by occupation. Thus, a representative study including various occupations and lifestyles is required to ensure generalizability of the findings of this study.

Second, in this study, the exclusive computational method estimating sleep debt was introduced by summing the differences between the estimated and expected sleep duration. Although similar methods have been used to estimate sleep debt in other studies (the difference between self-reported sleep need and sleep duration on weekdays) [69,70], sleep debt computed using these methods may not reflect the neurobehavioral impairment caused by chronic sleep loss commonly observed in the experimental condition [71,72]. Initially, we adopted this definition with the aim of having participants plan their daily activities to improve the status of sleep insufficiency, but it could be possible that actual sleep debt was not accurately estimated. Therefore, a reliable method to estimate current sleep status should be developed and used in future studies. Especially, combining EMA techniques and machine learning methods is expected to provide reliable sleep measurements in daily life. For instance, a recent study reported that machine learning techniques could estimate daily sleep quality by using complex life data obtained from EMA questionnaires [73]. Collecting and integrating multidimensional information would be meaningful not only in understanding the covariant associations of sleep behavior with daytime symptoms but also for developing novel sleep measurements.

Finally, participants could identify the group they were assigned to because only the participants in the feedback group received feedback messages at 9 AM. Considering that physical symptoms were measured using self-report evaluations, EMA recordings were affected by cognitive biases such as the Hawthorne effect (the inclination of people who participate in an experimental study to change or improve their behavior only because it is being studied and not because of changes in the experimental stimulus). Optimizing the study design would help clarify this point. For example, by sending intervention messages that are not related to habitual sleep behavior to participants in the control group, we can determine whether the finding is based on a specific response to sleep feedback messages. In addition, implementing a microrandomized trial [74], which is equivalent to a within-individual randomized controlled trial, could also be helpful in investigating the effectiveness of the interventions. By randomizing whether feedback messages are sent, we could examine the causal relationships between sleep behaviors and daytime functions.

### Conclusions

We conducted an mHealth trial with office workers and demonstrated that objective push-type sleep feedback stabilizes sleep timing and improves physical symptoms at wake-up time. However, we did not find evidence of prolonged sleep duration, advanced sleep timing, or improved work performance. Future research should incorporate specific behavioral instructions intended to improve sleep duration and sleep timing with the current protocol and investigate behavioral instruction effectiveness by integrating and using multidimensional information collected as part of daily life.

## Appendix

Multimedia Appendix 1 Screenshots of the health care Internet of Things app.

Multimedia Appendix 2 Validity of the Sciencenet activity monitor.

Multimedia Appendix 3 Sleep hygiene guide.

Multimedia Appendix 4 Additional analyses of the statistical properties of sleep variables.

## Abbreviations

BDI-II: Beck Depression Inventory second edition
BLE: Bluetooth Low Energy
CI: credible interval
EMA: ecological momentary assessment
HIT: health care Internet of Things
HPQ: Health and Work Performance Questionnaire
IoT: Internet of Things
mHealth: mobile health
PSQI: Pittsburgh Sleep Quality Index
SPF: Sleep Probability Function
STAI: State-Trait Anxiety Inventory

## Data Availability

The participants of this study did not agree to their data being shared publicly; therefore, supporting data are not available.
