# Supplementary material for: The Effects of Objective Push-Type Sleep Feedback on Habitual Sleep Behavior and Momentary Symptoms in Daily Life: mHealth Intervention Trial Using a Health Care Internet of Things System
Source: JMIR Mhealth Uhealth. 2022 Oct 6;10(10):e39150. doi: 10.2196/39150 (PMC9585447; doi:10.2196/39150)
Supplement: Multimedia Appendix 1 [file mhealth_v10i10e39150_app1.doc]

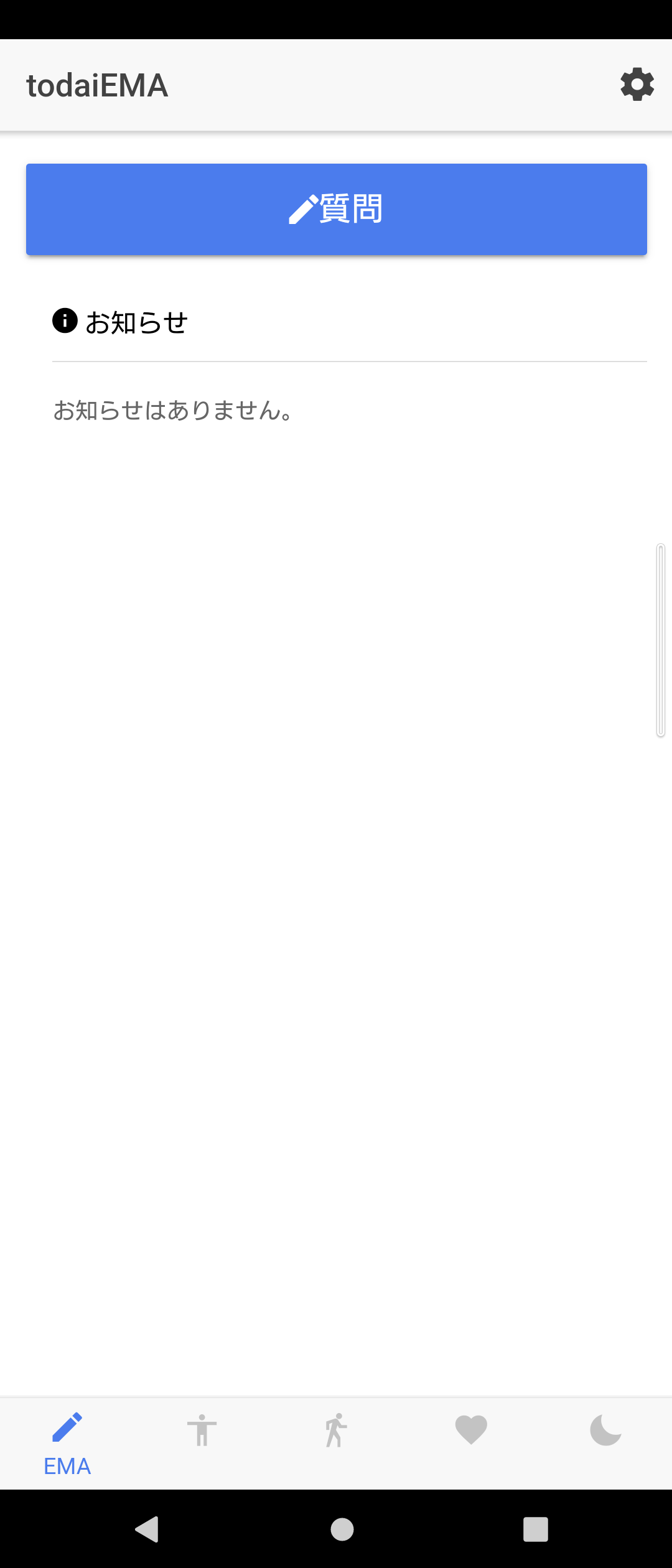
Multimedia Appendix 1: Screenshots of the HIT app

Multimedia Appendix 1, Figure 1: Home screen of the HIT app


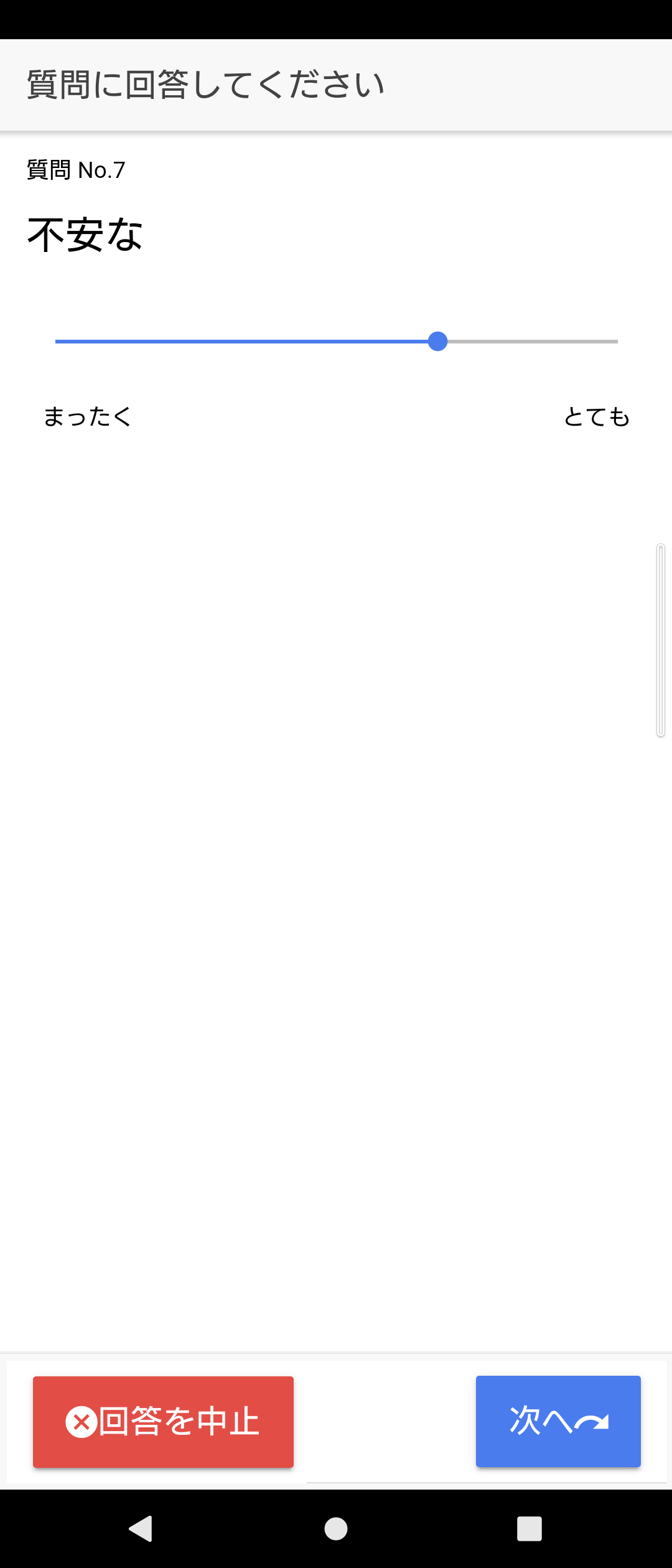


Multimedia Appendix 1, Figure 2: EMA screen of the HIT app

By tapping the [質問] icon on the home screen, the users can answer the EMA questionnaires.

The EMA measurements were rated using a visual analog scale from 0 to 100 displayed on the screen.


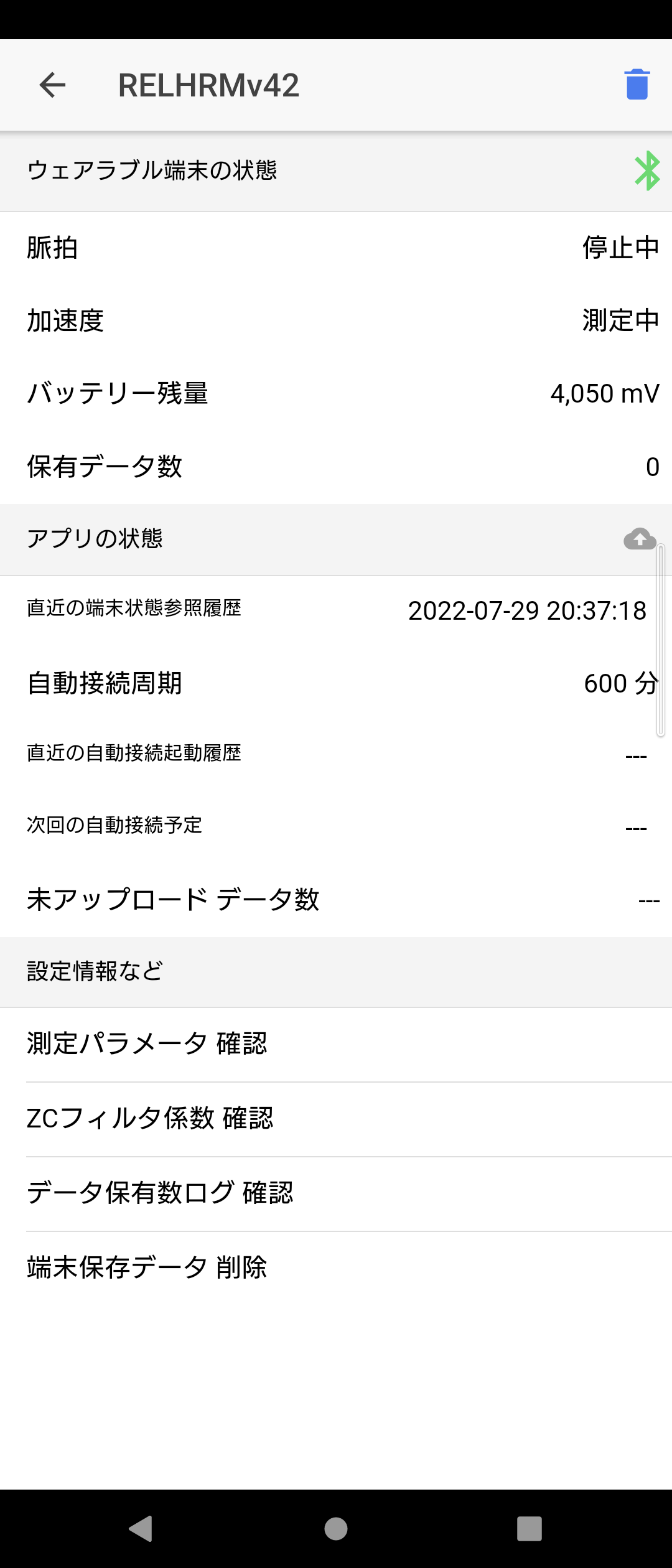


Multimedia Appendix 1, Figure 3: Pairing screen of the HIT app

The users can check the measurement status of the wearable activity monitor (stopping or recording) and battery status.


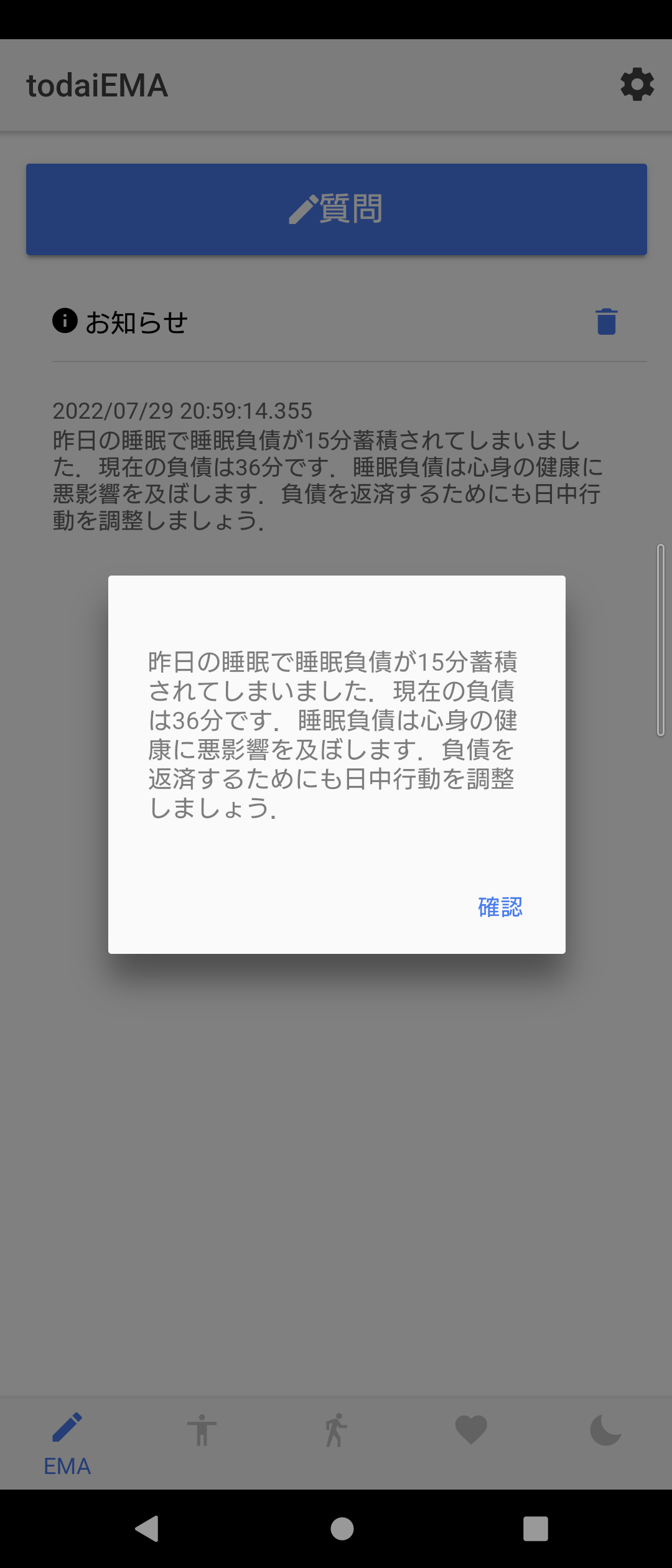


Multimedia Appendix 1, Figure 4: An example of personalized sleep feedback.

During the survey period, the personalized sleep feedback was generated based on objective measurements and popped up on the screen at 9 AM. This figure shows an example of the message when the user accumulated sleep debt; “You accumulated 15 minutes of sleep debt yesterday. Your current overall debt is 36 minutes. Sleep debt has adverse effects on physical and mental health. Adjust your daytime behavior to cancel your debt.”
