# Supplementary material for: The Effects of Objective Push-Type Sleep Feedback on Habitual Sleep Behavior and Momentary Symptoms in Daily Life: mHealth Intervention Trial Using a Health Care Internet of Things System
Source: JMIR Mhealth Uhealth. 2022 Oct 6;10(10):e39150. doi: 10.2196/39150 (PMC9585447; doi:10.2196/39150)
Supplement: Multimedia Appendix 2 [file mhealth_v10i10e39150_app2.doc]

Multimedia Appendix 2: Validity of Sciencenet activity monitor

To obtain the zero-crossing counts (ZC) data of the Sciencenet device, we first filtered the recorded body acceleration data with a 6th order Butterworth filter from 2 to 3 Hz. Thereafter, we counted the number of times the signal level crossed 0.01 G within an epoch for each axis. Finally, the largest value among the axes was used as the ZC data for the epoch.


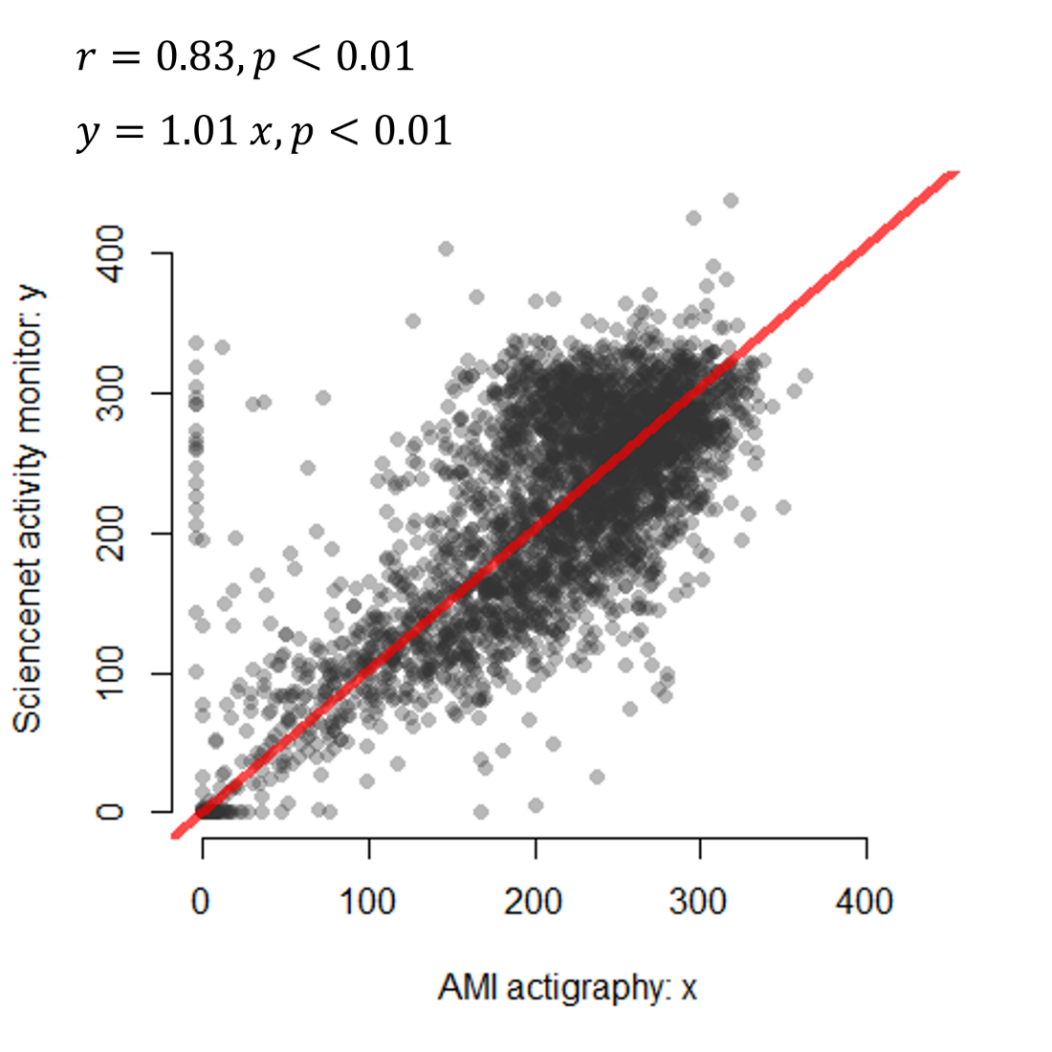


Multimedia Appendix 2 Figure 1. Scatter plot of ZC data.

The vertical and horizontal axes represent the ZC data of the AMI actigraphy and Sciencenet activity monitor, respectively. Data were recorded simultaneously from a specific participant for three days. We examined the validity of our in-house activity monitor using ZC data when the participant was awake. Correlation analysis showed a strong correlation between them (*r* = 0.83, *p* < 0.01), and the slope of the linear regression model was 1.01 (p < 0.01) when the intercept was assumed to be zero. These results indicate that the ZC data of our in-house device were comparable with those of the AMI actigraphy.

| Accuracy: 0.929  Sensitivity: 0.896  Specificity: 0.995 | | Sciencenet | |
| --- | --- | --- | --- |
|  |  | Wake | Sleep |
| AW2 | Wake | 3362 | 392 |
|  | Sleep | 9 | 1907 |

Multimedia Appendix 2 Table 1. Confusion matrix (in epochs) for sleep-wake identification.

We compared the performance of epoch-by-epoch sleep-wake identification between the AMI actigraphy and Sciencenet devices. The Cole-Kripke algorithm with Webster’s rescoring rules was adopted for the physical activity data of the Sciencenet device to identify whether the 1-minute epoch was sleep or wake. Thereafter, we used the same algorithm on the Action-W Version 2 software for analyzing the AMI actigraphy data. When comparing these outputs, the accuracy, sensitivity, and specificity were 0.929, 0.896, and 0.995, respectively.


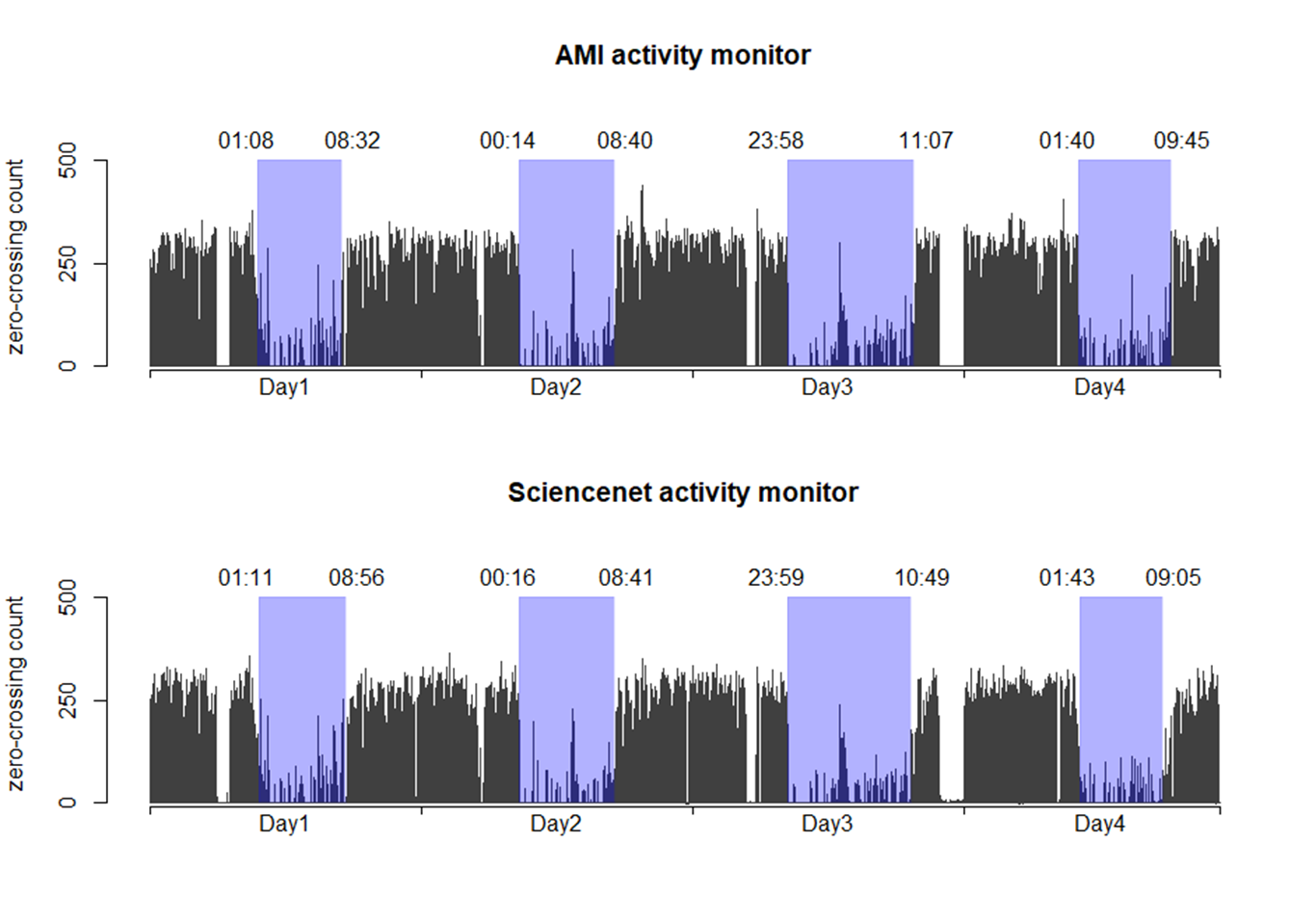


Multimedia Appendix 2 Figure 2. Representative data of estimated sleep intervals using the AMI and Sciencenet activity monitors.

The blue areas in the top and bottom panels indicate the estimated sleep intervals with Action-W Version 2 software and the dedicated Sciencenet algorithm, respectively. The figure indicates that the Sciencenet device and algorithm are analogous in performance to research-grade actigraphy.
