# Supplementary material for: The Effects of Objective Push-Type Sleep Feedback on Habitual Sleep Behavior and Momentary Symptoms in Daily Life: mHealth Intervention Trial Using a Health Care Internet of Things System
Source: JMIR Mhealth Uhealth. 2022 Oct 6;10(10):e39150. doi: 10.2196/39150 (PMC9585447; doi:10.2196/39150)
Supplement: Multimedia Appendix 3 [file mhealth_v10i10e39150_app3.doc]

Multimedia Appendix 3: Sleep hygiene guide

睡眠衛生ガイド

(Sleep hygiene guide)

・夕方までに、30分以上歩きましょう。

(・Walk for at least 30 minutes in the evening)

・規則正しい食生活をして、すきっ腹で寝ないようにしましょう。

(・Eat regularly and avoid sleeping with an empty stomach)

・夜に水分を取りすぎないようにしましょう

(・Do not drink too much water at night.)

・夕方以降は、カフェインの入ったものは避けましょう。

(・Avoid caffeinated drinks and foods in the evening)

・アルコールを避けましょう。

(・Avoid drinking alcohol.)

・就寝前の喫煙を避けましょう。

(・Avoid smoking before bedtime)

・昼休みに、30分以内の仮眠をしましょう。

(・Take a nap for 30 minutes during your lunch break.)

・始業時刻を調整して、睡眠時間を確保しましょう。ただし、決まった時刻に起床するようにしましょう。

(・Adjust your working hours to get more sleep. However, be sure to get up at a fixed time.)

・１時間に１回程度、立ち上がってストレッチをしましょう。

(・Stand up and stretch every hour)

・飲み物は、1回分だけ準備して、必要時に毎回歩いて取りに行きましょう。

(・When you want to drink water at work, stand up and walk to get it; prepare only one drink at a time.)
