# Supplementary material for: The Effects of Objective Push-Type Sleep Feedback on Habitual Sleep Behavior and Momentary Symptoms in Daily Life: mHealth Intervention Trial Using a Health Care Internet of Things System
Source: JMIR Mhealth Uhealth. 2022 Oct 6;10(10):e39150. doi: 10.2196/39150 (PMC9585447; doi:10.2196/39150)
Supplement: Multimedia Appendix 4 [file mhealth_v10i10e39150_app4.doc]

Multimedia Appendix 4: Additional analyses of the statistical properties of sleep variables


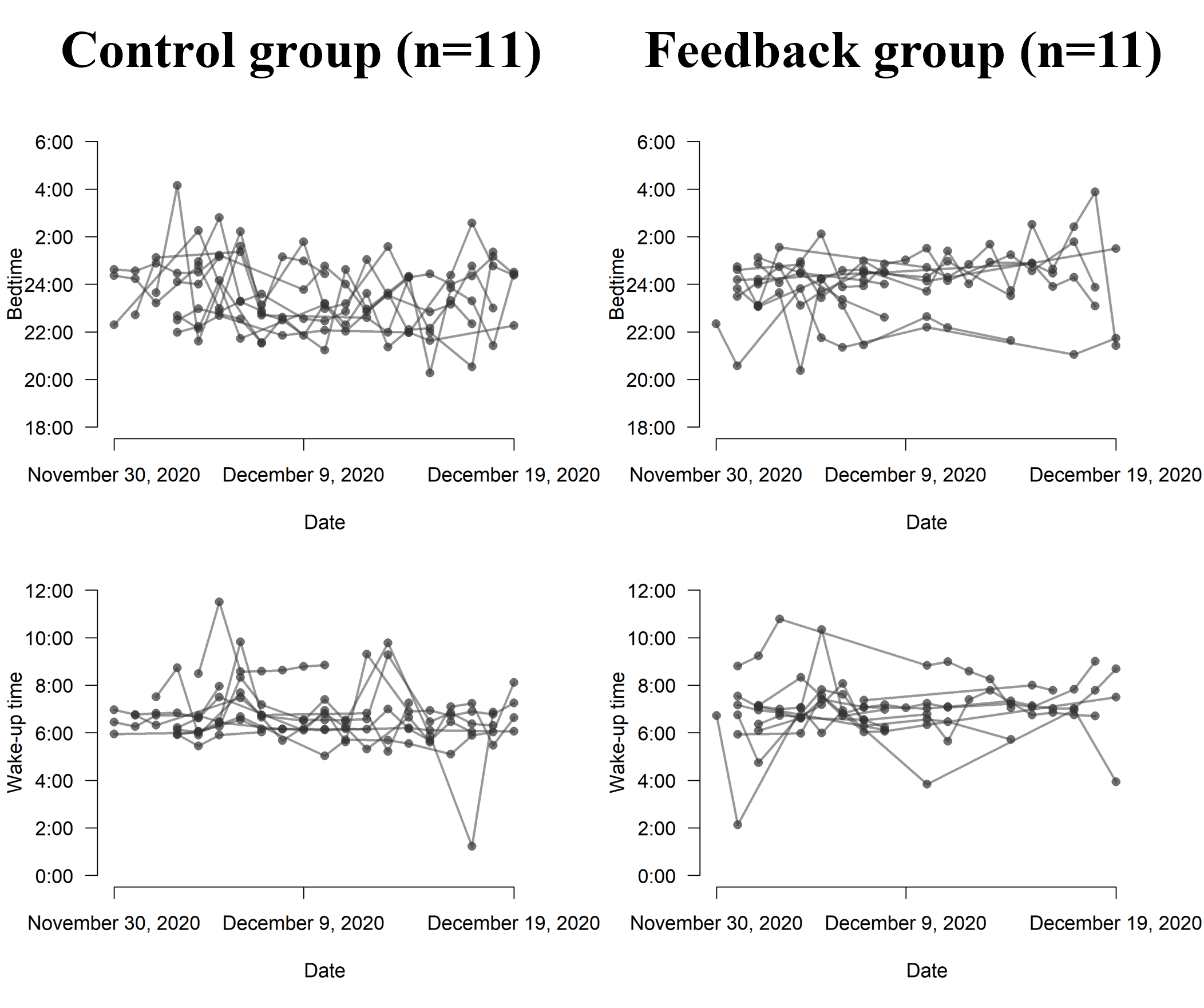


Multimedia Appendix 4 Figure 1. Spaghetti plots of estimated bedtime (top row) and wake-up time (bottom row) per participant for the trial period. The left and right columns indicate the time series of the sleep variables for the control and feedback groups, respectively.

Multimedia Appendix 4 Table 1. Results of the hierarchical Bayesian model for bedtime and wake-up time

|  | Control | | Feedback | | Difference | |
| --- | --- | --- | --- | --- | --- | --- |
|  | EAP^a^ (*SD*)^b^ | 95% CI^c^ | EAP (*SD*) | 95% CI | EAP (*SD*) | 95% CI |
| Bedtime^d, e^ | | | | | | |
| Intercept | 23:48 (27 min) | 22:55 to 24:41 | 23:49 (33 min) | 22:46 to 24:55 | -1 min (31 min) | -1 h 0 min to 1 h 3 min |
| Day | -1 min (1 min) | -4 min to 1 min | 1 min (1 min) | -1 min to 4 min | 3 min (2 min) | -1 min to 6 min |
| σ^(0)f^ | 52 min (11 min) | 34 min to 1 h 18 min | 52 min (11 min) | 34 min to 1 h 18 min | ― | |
| σ^(Y)^ | 1 h 19 min (5 min) | 1 h 9 min to 1 h 30 min | 1 h 0 min (5 min) | 52 min to 1 h 11 min | *-18 min* (*7 min*) | *-31 min to -4 min* |
| Wake-up time | | | | | | |
| Intercept | 7:17 (22 min) | 6:34 to 7:59 | 7:20 (27 min) | 6:27 to 8:12 | -5 min (6 min) | -17 min to 7 min |
| Day | -1 min (1 min) | -4 min to 1 min | 1 min (1 min) | -1 min to 3 min | 2 min (2 min) | -1 min to 5 min |
| σ^(0)f^ | 41 min (10 min) | 25 min to 1 h 4 min | 41 min (10 min) | 25 min to 1 h 4 min | ― | |
| σ^(Y)^ | 1 h 2 min (4 min) | 55 min to 1 h 11 min | 57 min (5 min) | 49 min to 1 h 7 min | -5 min (6 min) | -17 min to 7 min |

^a^EAP: expected a posteriori,

^b^SD of the posterior distribution.

^c^CI: credible interval.

^d^The models were run by controlling for age and sex.

^e^Italicized texts denote statistically significant group effects; at a 95% CI.

^f^The difference in the interindividual variability for the intercept (σ^0^) was not computed because σ^0^ was assumed to be equal between groups.
